# Laboratory Evaluation of a Robotic Operative Microscope - Visualization Platform for Neurosurgery

**DOI:** 10.7759/cureus.3072

**Published:** 2018-07-30

**Authors:** Evgenii G Belykh, Xiaochun Zhao, Claudio Cavallo, Michael A Bohl, Kaan Yagmurlu, Joseph L Aklinski, Vadim A Byvaltsev, Nader Sanai, Robert F Spetzler, Michael T Lawton, Peter Nakaji, Mark C Preul

**Affiliations:** 1 Neurosurgery, Barrow Neurological Institute, St. Joseph's Hospital and Medical Center, Phoenix, USA; 2 Neurosurgery, Barrow Neurological Institute, St. Joseph's Hospital and Medical Center, Phoenix , USA; 3 Neurosurgery, Barrow Neurological Institute/St. Joseph's Hospital and Medical Center, Phoenix, USA; 4 Neurosurgery, Irkutsk State Medical University, Irkutsk, RUS; 5 Division of Neurological Surgery, Barrow Neurological Institute, Phoenix, USA

**Keywords:** microscope, exoscope, endoscope, visualization, fluorescence, fluorescein sodium, 5-ala, indocyanine green, robotics, virtual reality

## Abstract

Background

We assessed a new robotic visualization platform with novel user-control features and compared its performance to the previous model of operative microscope.

Methods

In a neurosurgery research laboratory, we performed anatomical dissections and assessed robotic, exoscopic, endoscopic, fluorescence functionality. Usability and functionality were tested in the operating room over 1 year.

Results

The robotic microscope showed higher sensitivity for fluorescein sodium, higher detail in non-fluorescent background, and recorded/presented pictures with color quality similar to observation through the oculars. PpIX visualization was comparable to the previous microscope. Near-infrared indocyanine green imaging 3-step replay allowed for more convenient accurate assessment of blood flow. Point lock and pivot point functions were used in dissections to create 3D virtual reality microsurgical anatomy demonstrations. Pivot point control was particularly useful in deep surgical corridors with dynamic retraction. 3D exoscopic function was successfully used in brain tumor and spine cases. Endoscopic assistance was used for around-the-corner views in minimally invasive approaches. We present illustrative cases highlighting utility and new ways to control the operative microscope.

Conclusion

Improvements of the robotic visualization platform include intraoperative fluorescence visualization using FNa, integrated micro-inspection tool, improved ocular imaging clarity, and exoscopic mode. New robotic movements positively assist the surgeon and provide improved ergonomics and a greater level of intraoperative comfort, with the potential to increase the viewing quality. New operational modes also allow significant impact for anatomy instruction. With the increasing number and complexity of functions, surgeons should receive additional training in order to avail themselves of the advantages of the numerous novel features.

## Introduction

Operative microscopes are an integral part of the surgical armamentarium. They have become so fundamental to the success of modern neurosurgical and other surgical specialty procedures that they nearly define the advent of modern technology-assisted surgery and certainly are requisite for a modern standard of care. Operative microscopes have become active platforms for the development of improved user-control interfaces and robotic systems. Milestones in this technological evolution include the transition from monocular to binocular vision, the ability to alter magnification without affecting the focal length, objective lenses allowing for a continuous adjustment of the working distance, improvements in illumination sources, introduction of adjustable multiaxial counterweight balancing, incorporation of frameless navigation for image-guided surgery, and intraoperative fluorescence techniques [1]. These advances have led to a terminology evolution towards appraisal beyond microscope and to “visualization platform”, as these developments provide significantly more functions than previous operative microscopes.

The goal of this study was to perform a comprehensive assessment of a new robotic visualization platform with novel user-control features and to compare its performance to the previous model of operative microscope. Neurosurgical robotic systems have been tested previously [2], including documentation of a self-navigating operative microscope [3-4]. Our laboratory and clinical investigations were focused primarily around four main areas: (1) control, robotic features and handling; (2) video recording and educational value; (3) hybrid visualization functionality; (4) intraoperative fluorescence visualization modules. Several of the new microscope platforms are incorporating some or many of the functions of the microscope platform we have assessed, evolving into more than mere operative microscopes. We did not have access to other brands of operative microscopes to assess previous and new model platforms. This technology evaluation serves only to compare the performance of a significant new operative visualization platform development with a previous version of a neurosurgical microscope in widespread use and is not an endorsement of operative microscopes from Carl Zeiss AG. The systems evaluated in this study were FDA-approved for clinical use and not in "beta" version testing. Further refinements are projected for the newer microscope system (e.g., with surgical navigation system optimization) as greater widespread use is encountered and with surgeon feedback. Thus, we aimed this investigation to be an evaluation in terms of functions, rather than an individual brand or system.

## Materials and methods

Surgical visualization

**Figure 1:**
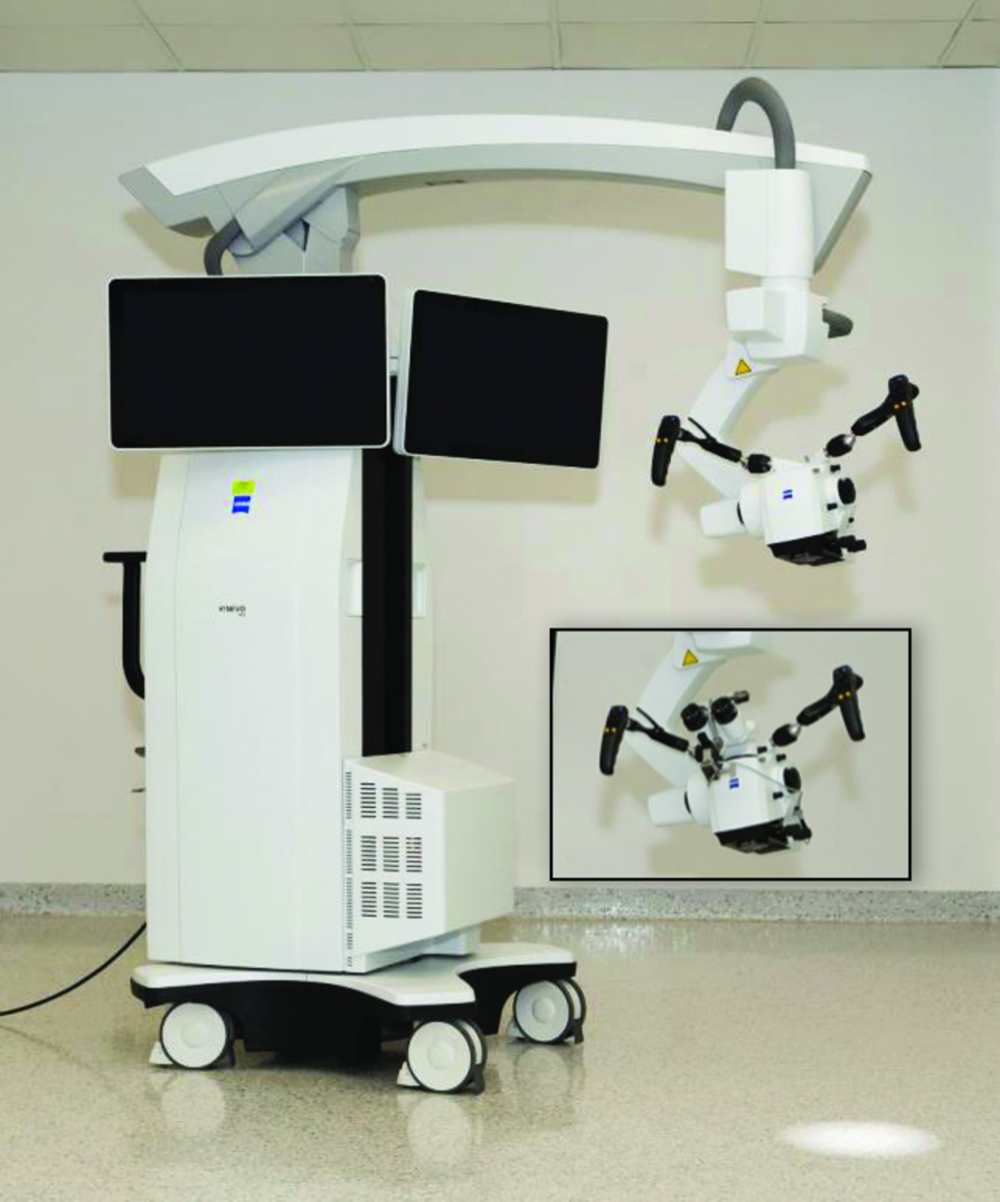
The robotic visualization system Illustration of the robotic visualization system ZEISS KINEVO 900 (Carl Zeiss AG, Oberkochen, Germany) (Used with permission from Barrow Neurological Institute, Phoenix, Arizona)

The new robotic visualization platform, ZEISS KINEVO 900 (Kinevo) (Figure 1) and the previous generation ZEISS PENTERO 900 (Pentero) (Carl Zeiss AG, Oberkochen, Germany) were evaluated in a neurosurgery research laboratory with experience in development and evaluation of operative visualization systems. We performed anatomical dissections in order to simulate surgical approaches on three formalin-fixed cadaveric heads with silicone vascular (i.e., arterial and venous systems) injection. The utility of control and robotic functions were subjectively assessed. We also assessed an exoscope option when viewing an image on a three-dimensional display through polarizing glasses (55’ screen size, 3D, 4K, model LMD-X550MT, Sony Corp., Tokyo, Japan). 3D projection employed passive linear light polarization technology that provided a perception of depth. In addition, we evaluated the QEVO, a 45-degrees viewing endoscopic micro-inspection tool integrated into the Kinevo designed to assist microsurgical procedures.

Interactive 3D virtual reality

Images acquired with the Kinevo were generated into interactive 3D virtual reality (3DVR) images using Object2VR software (Garden Gnome Software e.U., Vienna, Austria). 3DVR images were created for the cadaveric specimens to demonstrate and review anatomical landmarks within surgical approaches. A desktop workstation was used for editing (128 GB RAM, CPU Intel Xeon E5-2630 V3 @ 2.40 GHz (two processors), NVIDIA Quadro M4000 graphic card, Windows 7 professional, 64-bit operation system). The steps for creating 3DVR images are summarized in Table 1.

**Table 1:** Steps for the creation of the 3D interactive virtual reality files using the Kinevo * Steps 3-7 may be repeated for different layers during dissection focusing on the same target point to generate step-by-step guides

Step	Description
	Set microscope 3D recording as side-by-side view
	In microscope settings go to “Stand” and set “Settings Speed (XY)” in “Microscope”, “Point Lock” and “Position Memory” domains at the value of 20
	Position the microscope at a point of interest and lock the target using the “PointLock” function at the selected target.
	Using foot pedal joystick while “PointLock” function is on bring the microscope to the most left desired position
	Start recording
	Using the foot pedal joystick move the microscope head to the most right desired position
	Stop recording. This video fragment will be used to create one horizontal row of frames. *
	Transfer the video to the computer workstation.
	In the Sony Vegas software, extract the individual frames from the video using the “Render as Picture” function. The number of images could be as many as 60 per second, but a higher number of images would increase the final 3DVR file size and slow the presenting speed.
	Select two or three images for every second (keep 1^st^ and 31^st^ images or 1^st^, 21^st^ and 41^st^ images for each second) while deleting the other images to create a list of images for further use.
	Create interactive VR using the Object2VR. Use the resulting images from step 10 as an input. The grid size should correspond to the number of images used. The output file format can be .swf, .html5 or .mov files.
	The .swf files can be viewed in Internet Explorer.

Animal experiments 

Experimental procedures were performed according to the guidelines and regulations of the National Institutes of Health for the Care and Use of Laboratory Animals and approval from the St. Joseph’s Hospital and Medical Center Institutional Animal Care and Use Committee. We used 10-12 week-old female C57BL/6-luc2 mice and Wistar rats from The Jackson Laboratory (Bar Harbor, ME, USA). Surgeries were performed under ketamine and xylazine anesthesia for mice and a ketamine, xylazine, and acepromazine cocktail for rats.

Glioma Model

We used mice 20-30 days after orthotopic implantation with GL261-luc gliomas. 5-aminolevulinic acid (5-ALA), (200 mg/kg) was administered intraperitoneally two hours prior to surgery. Fluorescein sodium (FNa) (5 mg/kg) was injected intravenously 30 min before tumor imaging. Fluorescence was visualized using appropriate filters intraoperatively after craniotomy.

Microvascular Anastomosis Model

Rat groin and cervical regions were dissected to expose carotid and femoral arteries and to perform microvascular anastomoses. The surgery was performed under the exoscope viewing at the 3D monitor positioned in front of the surgeon. Intraoperative angiography was performed with indocyanine green (ICG) (0.5 mg/kg) to assess blood flow and appraise anastomosis quality.

Fluorescent phantoms

FNa dissolved in water was used in ex vivo experiments to compare the sensitivity of the green-yellow fluorescent visualization option of the platforms.

Tris (dibenzoylmethane) mono (1,10-phenanthroline) europium (lll) (#538965 Sigma-Aldrich, St. Louis, MO, USA) solutions (Eu) were used ex vivo to compare the sensitivity of the platforms to the red fluorescence. Eu has an excitation maximum at 355 nm and emission maximum at 615 nm, which is similar to protoporphyrin IX (PpIX), but Eu is more photostable.

Fluorescence visualization

Appropriate filters were used for intraoperative fluorescence visualization: YELLOW 560 for FNa, BLUE 400 for PpIX or Eu, and INFRARED 800 for ICG. Two Pentero microscopes (equipped with YELLOW 560/INFRARED 800 and BLUE 400/INFRARED 800) and one Kinevo (equipped with all three filters) were used. Settings were similar values for focal distance, magnification, tube length, light intensity and diaphragm diameter to ensure correct and unbiased comparison.

Image acquisition and analysis

Images were acquired by the internal cameras of the Kinevo and Pentero. In order to approximate and compare fluorescence visualization by an unaided surgeon’s eye through the oculars, we also acquired images with an iPhone 6s (Apple, Cupertino, CA, USA) through the oculars and microscope-mounted digital single-lens reflex camera (Canon USA, Inc., Melville, NY, USA). Images were processed for green, blue and red channels separation and intensities were analyzed in ImageJ (NIH) after drawing regions of interest over the fluorescent areas. A t-test was used for comparisons between groups with P<0.05 selected as a threshold for significance.

Clinical assessment

The usability and functionality of Kinevo with the QEVO endoscopic micro-inspection tool was tested in the operating room over nearly one year (2017). A patient study protocol was approved by the Institutional Review Board of the Barrow Neurological Institute. Patients signed a voluntary-informed consent form to participate in this study.

## Results

Robotic features

**Figure 2:**
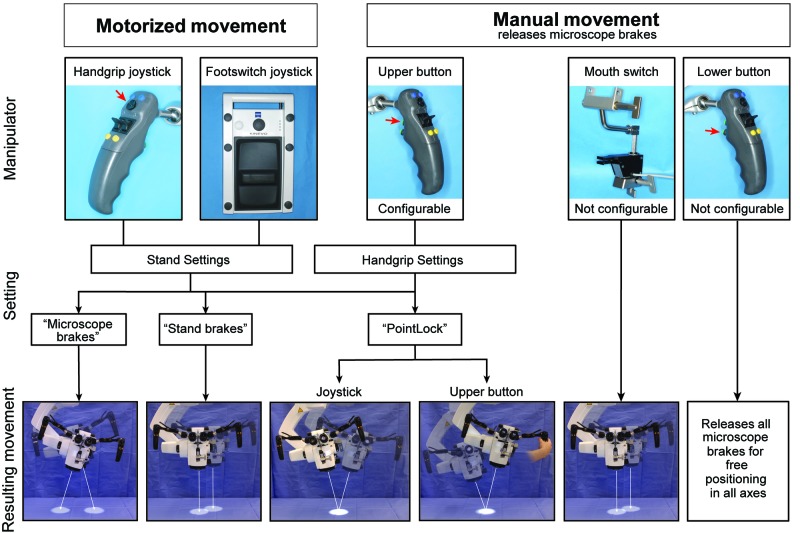
Manipulations and Movements Schematic representation of the possible ways to operate and move the Kinevo system. The movements can be generated by motors and by manual movements holding the handgrips. Menu settings allow selecting three possible functions separately for the joystick and for the upper button on the handgrip. (Used with permission from Barrow Neurological Institute, Phoenix, Arizona)

Possible means to operate and move the Kinevo system during surgery are systematized in Figure 2. There are essentially 2 ways to operate the microscope: manual mode (via brake buttons on the handgrips or mouthpiece) and motorized robotic mode (via a joystick on handgrips or foot control panel).

**Figure 3:**
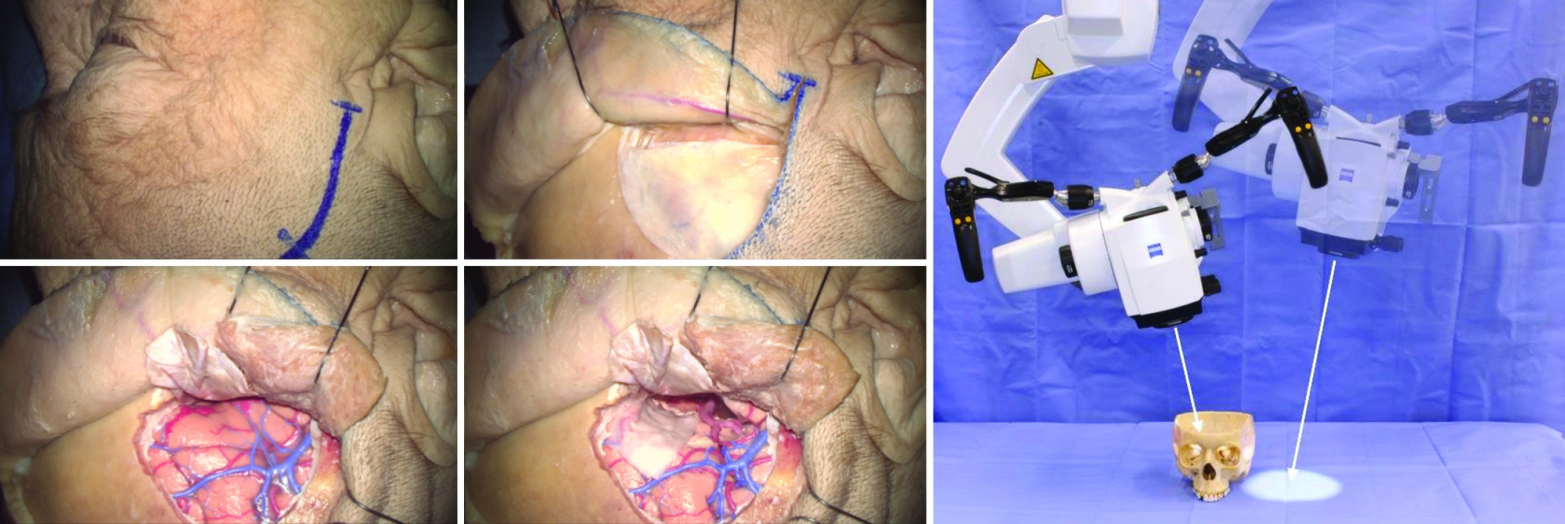
Position Memory Illustration of the “Position Memory” concept that allows the microscope to return to a preset position based on multiple spatial coordinates previously recorded during the surgical procedure. (Used with permission from Barrow Neurological Institute, Phoenix, Arizona)

The “PositionMemory” function enabled the robot’s position, trajectory, focus point, and magnification to be returned to previously saved positions at any time during the dissection or surgical procedure (Figure 3). “PositionMemory” was useful in creating step-by-step views of relevant anatomy with minimal effort for platform repositioning. When the Kinevo moved to the desired location, it overlaid a semi-transparent image from the previous position that assisted with image alignment. Such near perfect alignment during or after repositioning was not possible during dissection or surgery with the Pentero.

**Figure 4:**
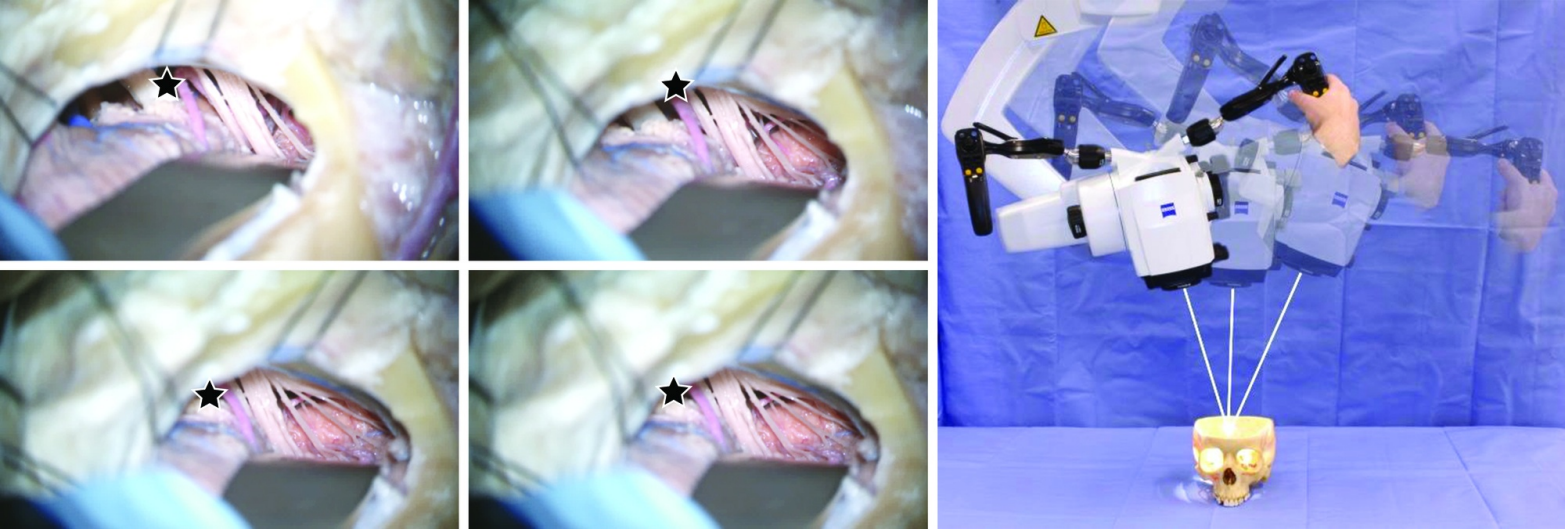
Point Lock Illustration of the “Point Lock” concept that allows pointing at a region of interest and locking the target while moving the microscope to different spatial positions. (Used with permission from Barrow Neurological Institute, Phoenix, Arizona)

**Figure 5:**
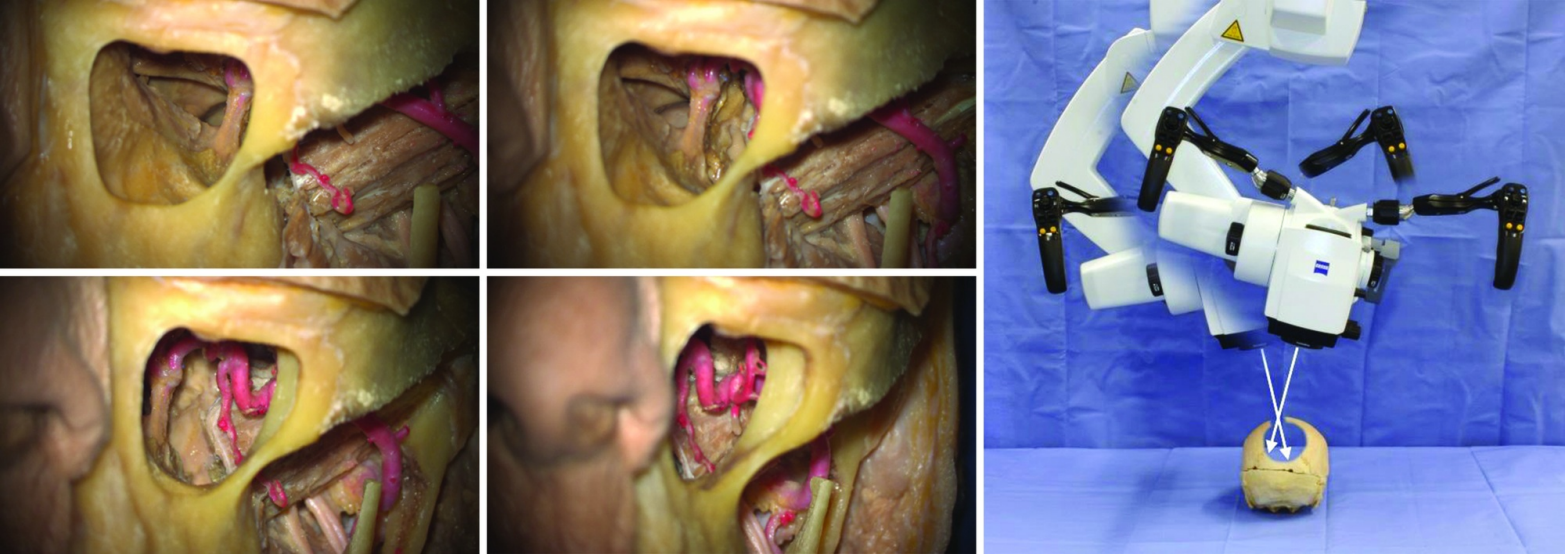
Point Lock for a Keyhole Illustration of the “PointLock” concept for a keyhole application. Visualization of the maxillary artery through a maxillary sinus by pivoting around previously selected superficial point on the bone window. (Used with permission from Barrow Neurological Institute, Phoenix, Arizona)

Motorized movements of the Kinevo could be selected in three options: “PointLock”, “Stand Breaks”, (movements parallel to surface) or “Microscope Breaks” (swiveling movements of the microscope head). When “PointLock” function was selected for the upper button of the handgrip, it released the brakes, allowing the manual movements of the microscope head while remaining in automatically adjustable focus and angle towards targeted point. Similarly, when “PointLock” function was active, joysticks moved the microscope head (motorized movement) in 4 orthogonal directions on a sphere (Figure 4). Compared with the Pentero, in which the motorized movements are not available, the new “PointLock” function allows visualization of a selected anatomical region from multiple different angles with a constantly focused picture, which is especially useful when operating through a key-hole surgical approach (Figure 5).

3D exoscope

The maximal measured working distance was significantly longer for the Kinevo (657 mm at 0.9-5.2x (range) magnification), compared to the Pentero (513 mm at 1.2-6.6x magnification). A 144-mm increase in working distance allowed exoscopic head positioning above the surgeon’s line of view to the monitor. We performed end-to-side anastomoses on five rat carotid arteries confirming the feasibility of the 3D exoscope to visualize fine surgical details. The Kinevo was used as an exoscope in 10 clinical cases. It was noted during these cases that a significant learning curve exists for becoming accustomed to operating with exoscope visualization. Most neurosurgeons today are accustomed to operating through an operative microscope, although, the exoscope provides a very different feel than a standard microscope and thus requires accommodation and practice. However, once the surgeon has become accustomed to operating while looking at a 3D projection of the surgical site away from the actual surgical site, the exoscope provided additional freedom of movement, easy positioning and target finding, and greater degrees of surgical freedom.

Intraoperative fluorescence

FNa Fluorescence under the YELLOW 560 Filter

**Figure 6:**
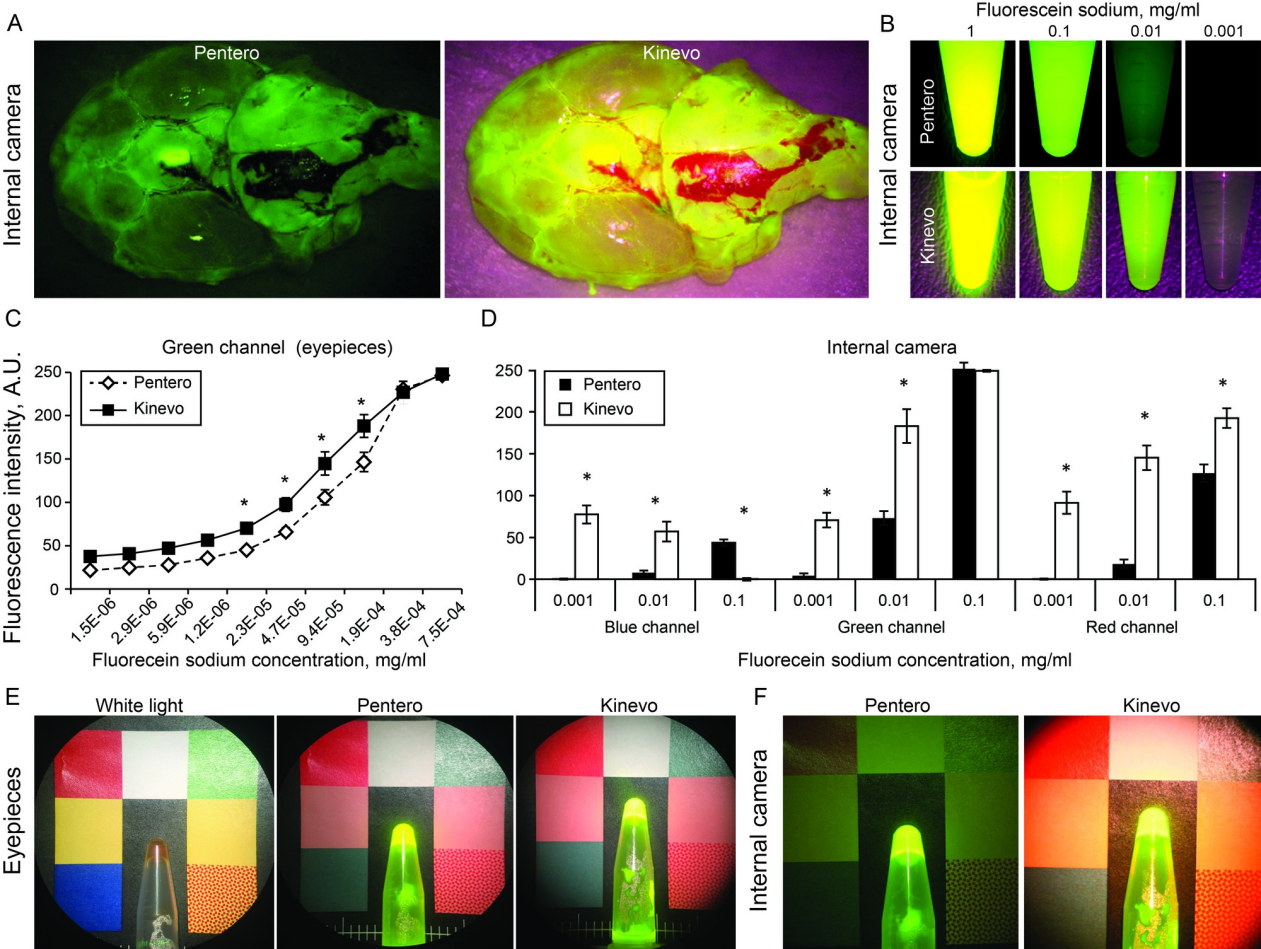
Laboratory experiments with fluorescein sodium (FNa) visualization A: Normal rat brain after FNa injection visualized using internal camera and YELLOW 560 by Pentero and Kinevo. B: Comparison of the internal camera views of the vials with various concentrations of FNa. C: Graph showing the fluorescence intensity against FNa concentration recorded by the two systems. The function demonstrates a significantly higher sensitivity of the Kinevo camera recordings for lower FNa concentrations. D: Comparison of signal intensities in blue, green, and red channels respectively. FNa signal detection is significantly increased in the green channel of the Kinevo. Differences in the signal intensities in blue and red channels reflect the optimized optical recording system allowing a brighter overall picture. E and F: Comparison of the oculars views (E) and internal camera views (F) of the vial with FNa positioned on a colored background *- t-test p<0.0001. (Used with permission from Barrow Neurological Institute, Phoenix, Arizona)

**Figure 7:**
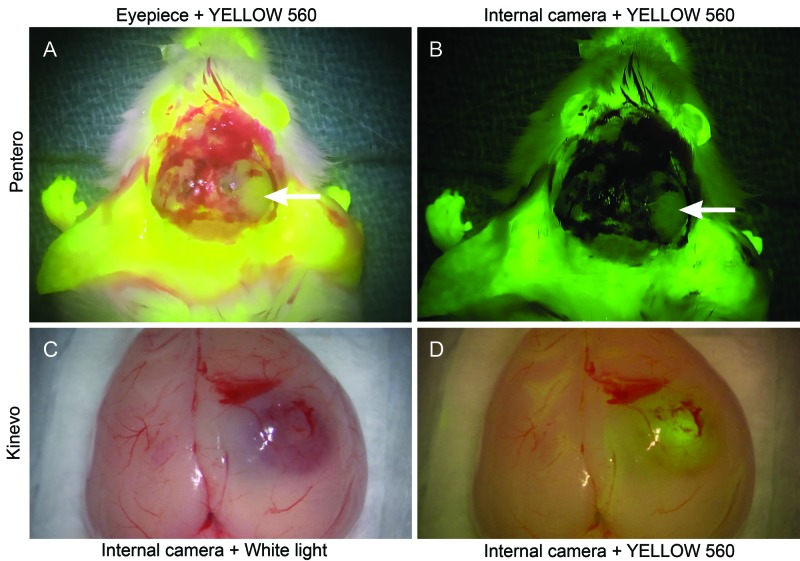
Illustration of a rat brain harboring a tumor after intravenous FNa injection. A: Picture taken through the oculars of the Pentero microscope under YELLOW 560 mode simulates perception by the unaided eye through the oculars. B: The Pentero internal camera with YELLOW 560 mode. The field appears much darker and the fluorescent tissue appears green in color compared to the bright yellow colors in the oculars. C: The Kinevo internal camera under white light illumination. D: The Kinevo internal camera under YELLOW 560 mode demonstrating only a subtle darker shade of normal brain compared to the white light illumination and a bright yellow fluorescence of the tumor. (Used with permission from Barrow Neurological Institute, Phoenix, Arizona)

Imaging of FNa phantoms revealed comparable fluorescence intensities through the oculars of the Kinevo and the Pentero (Figure 6). However, the Kinevo internal video camera demonstrated better sensitivity for detecting lower FNa concentrations and also for visualizing nonfluorescent tissue. This was especially prominent during imaging of murine brains with and without gliomas after FNa injection. Although views through the oculars were similar between the Pentero and the Kinevo, images acquired with the internal cameras showed significantly more contrast using the Kinevo compared to the Pentero. The Kinevo internal camera recordings had a similar color quality to those perceived by a human unaided vision in the oculars (Figure 7). This observation was also similar when the Kinevo was used in an exoscopic 3D mode. Muscle color appeared as a more raspberry tan hue with the Kinevo, while the same tissue appeared a darker hue brown with the Pentero.

PpIX and Eu Fluorescence under the BLUE 400 Filter

**Figure 8:**
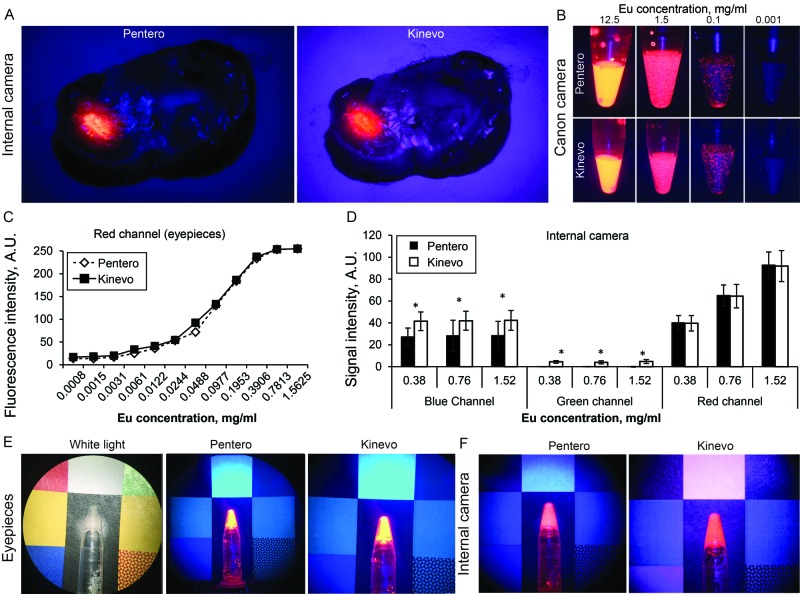
Laboratory experiments with red fluorescence signal visualization A: Mouse brain with tumor two hours after 5-ALA injection visualized using internal cameras and BLUE 400 mode by the Pentero and the Kinevo. B: Comparison of ocular views of the vials with various concentrations of Europium (Eu). C: Graph of the fluorescence intensity against Eu concentration demonstrates a similar sensitivity of the two systems as recorded through the oculars. D: Comparison of the signal intensities of the Eu dye standards in blue, green, and red channels respectively as recorded through the oculars. Signal intensity in the red channel is similar in the Kinevo and the Pentero. Differences in the signal intensities in green and red channels reflect the optimized optical imaging system in the Kinevo allowing a brighter overall picture in BLUE 400 mode. E and F: comparison of the ocular views (E) and internal camera views (F) of the vial with Eu positioned on a colored background *- t-test p<0.0001. (Used with permission from Barrow Neurological Institute, Phoenix, Arizona)

Observations of PpIX and Eu fluorescence showed no significant differences when visualized on the Kinevo or the Pentero using the oculars or internal video camera (Figure 8). Eu signal from the red channel was similar between the Pentero and the Kinevo as viewed with the oculars and internal camera. However, intensities in blue and green channels were slightly but significantly higher with the Kinevo for the internal camera. The colors recorded by the Kinevo internal camera appeared more pinkish than in the Pentero, making the surrounding non-fluorescent brain tissue brighter and more visible.

Fluorescence under the INFRARED 800 Filter

The infrared mode of both systems included 3-step video replays of ICG videoangiography. The first step entailed the video recording process, the second step provided a replay of a shortened version of the video, and the third step was a full-length replay of the recorded videoangiography. Using the Kinevo exoscope we visualized in sharp detail small surface vessels of the rat brain and assessed with clarity the consistency of the microvascular anastomosis of rat carotid arteries.

**Figure 9:**
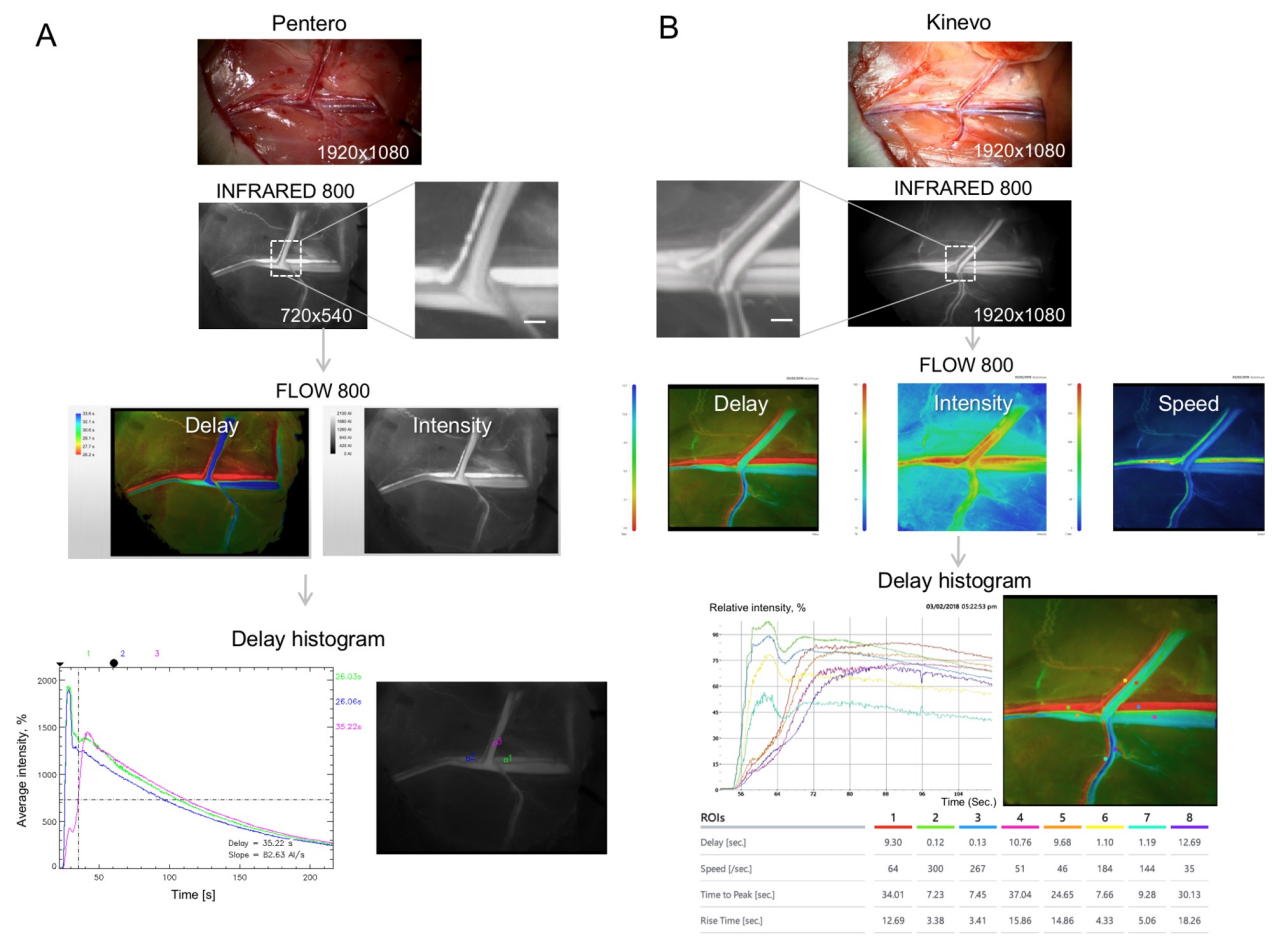
Laboratory experiments with indocyanine green (ICG) and FLOW 800 The figure shows a workflow of (A) the Pentero and (B) the Kinevo INFRARED 800 modes. ICG angiography (0.5 mg/kg IV) was performed after exposure of the left femoral vascular bundle of a rat. FLOW 800 functions can display colored maps of contrast delay and average intensity. Additionally, the Kinevo can display a colored map of relative speed values. The Kinevo has robust video-editing functions, which simplifies flow assessment.  After selection of regions of interest, the histogram analysis can be performed on both microscopes to obtain exact values of delay (s), speed (1/s), time to peak (s), and rise time (s). Scale bar = 1 mm (Used with permission from Barrow Neurological Institute, Phoenix, Arizona)

FLOW 800 analyses showed similar quantitative results for rat femoral vessels in both systems as images reveal for “Delay” and “Intensity” and histogram of “Delay” (Figure 9). However, the Kinevo presented a new additional function of a colored “Speed” map that displayed a precise outline of the rat femoral vessels. The “Speed” map function demonstrated a more realistic image derived from the original INFRARED 800 image, conveying similar anatomic detail, but at the same time adding functional information in terms of blood flow speed. Histograms were created to perform a quantitative assessment of the contrast delay, average intensity and flow speed, for the selected regions of interest, which may be critical to assess hemodynamics in the brain vasculature.

Clinical experience

Usability and features of the Kinevo were assessed in 78 neurosurgical procedures (Table 2). In terms of the functions, 6 cases used “PositionMemory”, 9 cases used “PointLock”, 10 cases used exoscope mode, and 19 cases used the endoscopic micro-inspection tool. The use of robotic functions was inconsistent during the study since those functions were relatively new to the neurosurgeons. The micro-inspection endoscopic tool was used mainly in tumor or aneurysm surgeries to check for tumor residual in the obscured regions (i.e., sides) of the resection cavity or after aneurysm clipping. ICG angiography was used in the majority of vascular cases including 4 arteriovenous malformations (AVM), 16 aneurysms, and 4 bypasses and was subjectively equally effective to the similar mode on Pentero. YELLOW 560 was used in 9 cases, including 6 tumors, and in 3 vascular applications for an AVM and 2 aneurysms. We present two clinical examples to illustrate some of the convenient new features of the Kinevo.

**Table 2:** Surgical cases performed with using the Kinevo microscope AVM – arteriovenous malformation, MVD – microvascular decompression

Lesion type	Number of cases
Total	78 (100%)
Intracranial tumors	29 (37.2%)
Vascular	
Aneurysm	21 (27.0%)
AVM	6 (7.7%)
Cavernous malformation	4 (5.1%)
Microvascular bypass	6 (7.7%)
Endarterectomy	3 (3.8%)
Spine fusion	6 (7.7%)
MVD	2 (2.6%)
Intracerebral hemorrhage	1 (1.3%)
Functions used	
Position Memory	6 (7.7%) (3 AVMs, 3 intracranial tumors)
Point Lock	9 (11.5%) (3 AVMs, 5 intracranial tumors, 1 aneurysm)
Exoscope	10 (12.8%) (8 intracranial tumors, 1 aneurysm, 1 endarterectomy)
Micro-inspection tool (QEVO)	19 (24.4%) (2 AVMs, 6 intracranial tumors, 4 aneurysms, 2 spine surgery, 1 bypass, 1 MVD, 1 endarterectomy, 2 cavernous malformation)
Fluorescence	
INFRARED 800	24 (30.8%) (4 AVMs, 16 aneurysms, 4 bypasses)
YELLOW 560	9 (11.5%) (6 intracranial tumors, 2 aneurysms, 1 AVM)

Case 1

**Figure 10:**
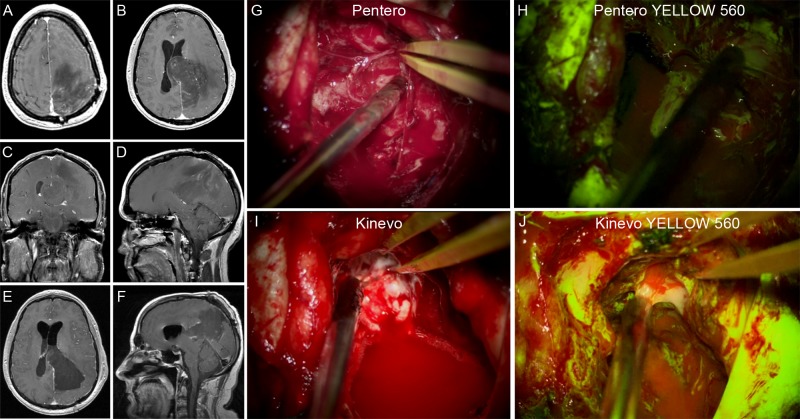
Illustrative case 1 Resection of the oligodendroglioma recurrence under the FNa fluorescence guidance. A-D: Preoperative T1 contrast enhanced MRI scans showed a left-sided parietal-occipital intra-axial lesion. E-F: Postoperative T1 contrast enhanced MRI scans showed a gross total resection. G: The Pentero display with white light. H: The Pentero display with YELLOW 560 mode. I: The Kinevo display with white light. J: The Kinevo display with YELLOW 560 mode (Used with permission from Barrow Neurological Institute, Phoenix, Arizona)

A 43-year-old male with a history of previously treated oligodendroglioma presented with progressive right-sided weakness. A large tumor recurrence was found on MRI (Figure 10). The patient underwent FNa-guided resection under the YELLOW 560 modality. Kinevo visualization showed clear, bright tumor fluorescence in contrast to the surrounding normal brain. Although the images were comparable in the oculars, on the monitor Kinevo provided significantly better illumination of the operative field than Pentero. Immediate post-operative radiological findings were consistent with a gross total resection. The patient recovered and his pre-operative symptoms resolved soon after surgery.

Case 2

**Figure 11:**
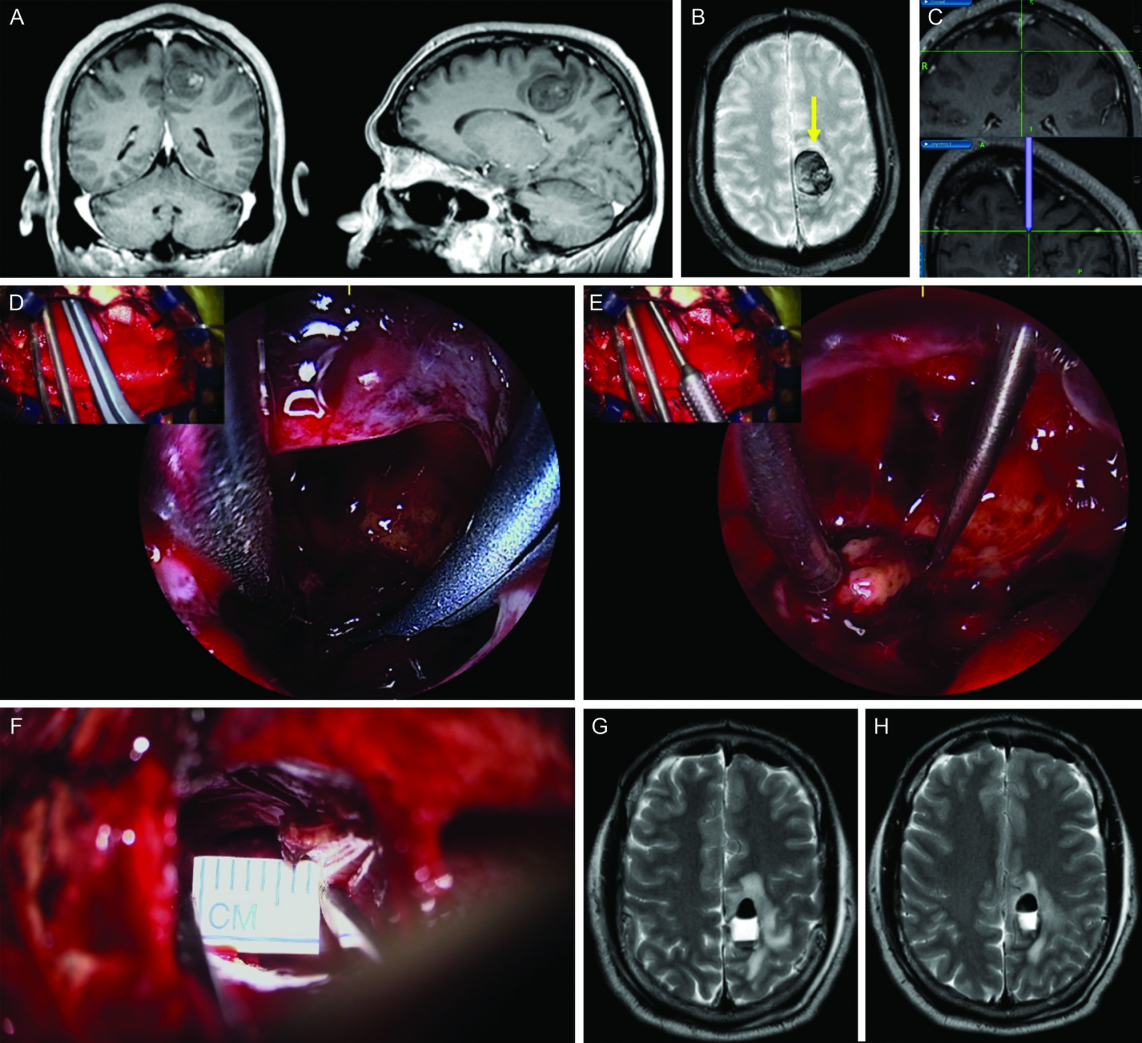
Illustrative case 2 The Kinevo endoscope-assisted microsurgical resection of the cavernous malformation. A-C: preoperative T1 contrast enhanced (A, B) and GRE (C) MRI scans showed a lesion near the motor strip in the medial posterior frontal lobe. D: Trajectory of the surgical approach. E-F: intraoperative assistance of the micro-inspection tool and the picture-in-picture feature. Microscopic view is shown in the top-left inset. G: Minimal corticotomy window was created for the approach. H-I: postoperative T2 MRI scans showed gross total resection. (Used with permission from Barrow Neurological Institute, Phoenix, Arizona)

A 47-year-old male presented with an acute right-sided weakness and was found to have multiple cavernous malformations on MRI (Figure 11). The left posterior frontal lesion located in close proximity to the motor strip was thought the most likely cause of recent symptoms. The patient underwent an endoscopic-assisted contralateral transfalcine interhemispheric approach with the use of the micro-inspection tool and the Kinevo. The micro-inspection tool allowed inspection of areas around the resection cavity. Several blind spots were identified using this tool which afforded the opportunity to resect hidden portions of the cavernous malformation. Post-operative imaging confirmed a gross total resection. The patient was back to full strength at 1 month after surgery.

## Discussion

Progress in Visualization

The surgical or operative microscope is intimately intertwined with the advancement of neurosurgical technique and operative technological capability. The co-development of operative microscopes and neuronavigation technologies has vastly expanded the neurosurgeon’s ability to safely and effectively treat lesions once thought unreachable [5]. As neurosurgeons continue pushing the limits of what is surgically achievable in hopes of affording better care for their patients, advances in operative microscope technologies become increasingly critical. Improved and specialized illumination, extreme stereoscopic visualization for the definition of small structures in constricted areas, smooth and rapidly responsive controlled movements, and the development of new technologies that afford increased visualization through minimally invasive approaches will continue to drive the field forward to achieving better outcomes [1]. Three key components in every neurosurgical procedure include the approach, the visualization, and the management of the target. Better vision can be acquired by optimal approach design and patient positioning; however, imaging clarity and illumination are dependent solely on the visualization tool.

The robotic auto-navigation system in the last generation of the Pentero microscopes was designed to improve intraoperative visualization and automatic positioning [4]. Several options have been introduced for such movement control [3-4] including Auto Lock Current Point, Align Parallel to Plan, and Point to Plan Target. The Auto Lock Current Point allows surgeons to lock on a surgical target allowing the microscope to rotate focus to the point in a new angle when manually moved to a new position; the Align Parallel to Plan positions the microscope to the preset angle and focus aligned to the planned target and trajectory, without the need to adjust the microscope when it is brought to the operative field; the Point to Plan Target automatically adjusts the focus on the predefined target. Such functions integrated into the operative microscope facilitate the focusing process while changing the angle of vision [1].

The Kinevo offers “Position Memory” and “Point Lock” functions that were not previously possible, except with the Elekta Surgiscope (Elekta Co., Stockholm, Sweden) or Zeiss MKM (Carl Zeiss AG, Oberkochen, Germany) systems, which are no longer produced [6-8]. “Point Lock” function guarantees a constantly focused vision at the fixed target when manually or automatically moving the microscope head during the surgery. This technology is thought to be helpful in the assistance of key-hole surgeries or procedures requiring small bone windows. In this way, intraoperative time may be significantly reduced, as there is no need to constantly adjust the parameters of the microscopic visualization during surgery. Considering that up to 40% of the total duration of surgery is spent in adjusting either the microscope’s position, viewing angle or focus [9], this technology could lead to decrease in operative time.

The entire operative room team can appreciate the detailed structures in the depth of the surgical corridor when observing the 3D projecting 4K monitor while operating under exoscopic visualization mode with long working distance (up to 657 mm at 5.2x magnification). The Kinevo has followed in incorporating exoscope visualization and surgeon-controlled robotic functions, similar to other available exoscopes recently introduced in the field [10]. During evaluation of the Kinevo as an exoscope with the 3D view, it was feasible to perform microvascular suturing and other microsurgical manipulations on live and cadaveric models. Neurosurgeons who use loupes may benefit from the magnified operative view on a 3D monitor while also able to observe the macroscopic picture by the naked eye. This situation was deemed especially relevant during cortical surface tumor and open spinal surgery cases. Furthermore, 3D widescreen exoscopic view increases educational value, as the whole operative team observes the same view.

Maneuverability

The robotic auto-positioning features for improved coordination with neuronavigation and automatic alignment to the designed plan were described previously [3, 4]. In contrast, here we assessed the “Position Memory” and “Point Lock” functions that are new. The system could smoothly and rapidly transit back to an indefinite number of positions, selected and saved intraoperatively. This feature may be useful when comparing two similar views at a different time during surgery, especially for repetitive FLOW 800 analysis. For instance, it may provide a different surgical perspective for clip repositioning after an unsatisfactory indication from ICG videoangiography or to illustrate or review steps of the surgical technique. The potential drawback inherent to all robotic and sophisticated computer controlled electronic systems is the possibility for movement or location-positioning errors, episodes of freezing, and unresponsiveness to the user. We have witnessed improvements in positioning accuracy with system's software updates during the course of the investigation.

Endoscopic micro-inspection tool

Endoscope assistance in microscope-operated intracranial surgeries offers an additional visualization of the deep structures. Previous quantitative comparative studies revealed that the endoscopic-assisted technique can achieve a greater exposure or viewable area than using conventional microscopic open access [11-12]. However, endoscope assistance is limited by a decrease or at least significant impediments in maneuverability when compared with using the operative microscope [13]. High-quality videos and feasibility in many neurosurgical scenarios have made the endoscope a popular additional visualization tool in neurosurgical practice.

**Figure 12:**
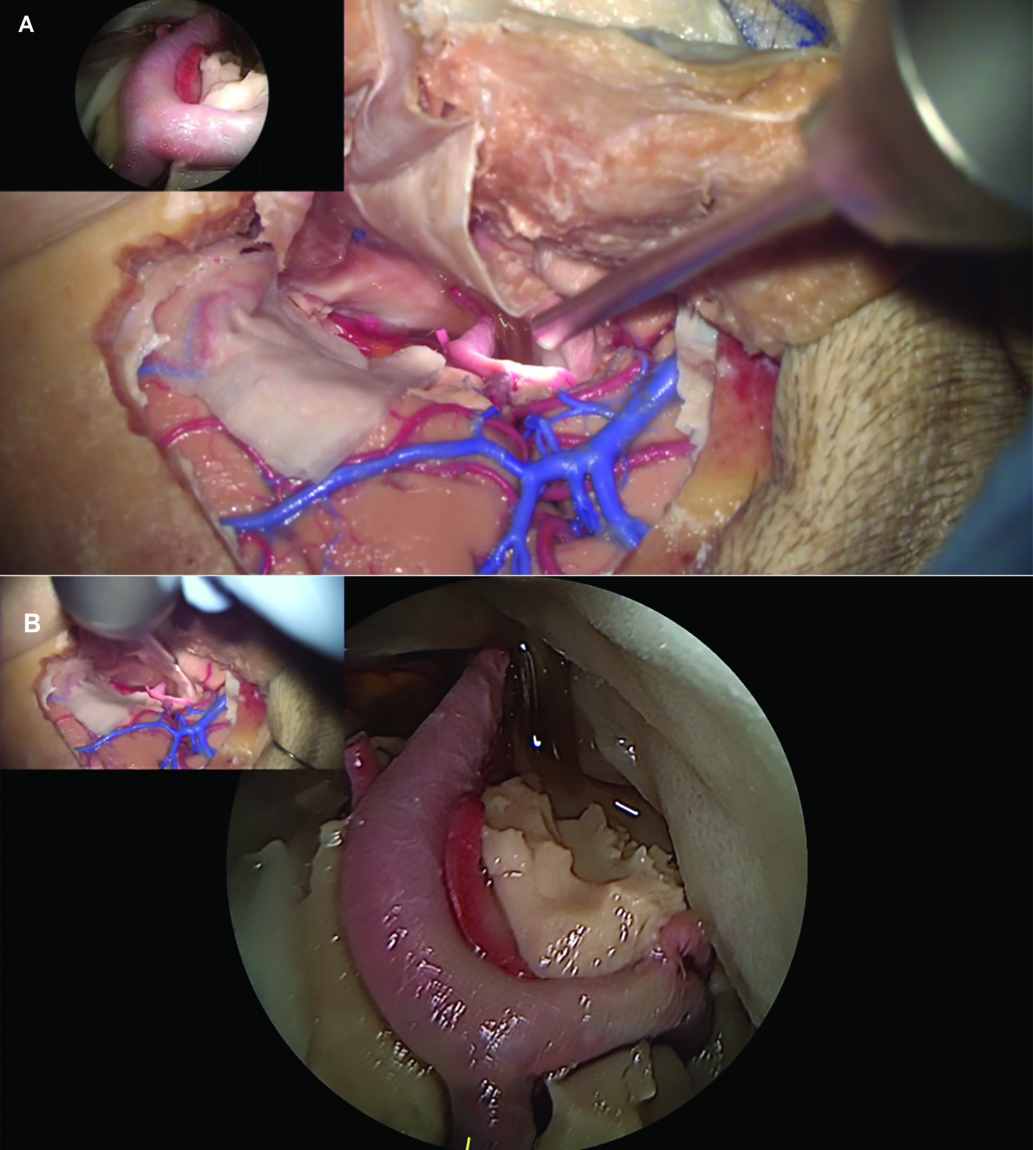
Picture-in-picture feature Illustration of the picture-in-picture feature of the Kinevo endoscopic micro-inspection tool. A: Microscopic view, the endoscope view is at the top-left; B: The endoscopic micro-inspection tool view, the microscopic view is at the top-left (Used with permission from Barrow Neurological Institute, Phoenix, Arizona)

Although the picture-in-picture concept that combines both endoscopic and microscopic view in one monitor was documented previously [14], the Kinevo is a visualization platform that solidifies integration of microscopic and exoscopic visualization with a micro-inspection angled endoscope. The combination of two views in a picture-in-picture format provides a safe and efficient way to perform endoscope assisted procedures (Figure 12). The exoscopic view covers the area behind the endoscope and would seem to allow safer and more control when inserting instruments into the surgical corridor.

Virtual reality for anatomical teaching and learning

Understanding complex volumetric relationships of neuroanatomical structures have been tremendously improved with stereoscopic or 3D recording and projection [15-17]. The development of virtual reality (VR) technology could provide further details in addition to traditional 2D illustration [18]. Three-dimensional videos are common in neurosurgery, however, the learning process is still a passive one. On the contrary, VR involves an active learning process and with an appropriate environment the learner could self-navigate the angle of approach and compare various views or trajectories to the desired target area.

Previously, we described a method to produce 3D Quick Time Virtual Reality (QTVR) of anatomic dissections [8]. However, this was performed with a robotic operative microscope (Zeiss MKM) with research laboratory hardware and software upgrades that did not achieve wide clinical use. In this study, we were able to demonstrate the use of the Kinevo for image acquisition and subsequent creation of a similar VR anatomy environment. Images were recorded with either the “inverted cone” or “key-hole” method using the “PointLock” function. 4K video recording produced images with quality comparable to the digital SLR cameras. Extracting frames from 4K videos also resulted in optimally rendered VR files. VR files could be produced in a two-dimensional mode or side-by-side 3D mode. The average length of video used was about 8 seconds, while the image extraction and processing took about 10 minutes for a single VR image production. A smooth transition of the Kinevo head at constant speed paired with fixed time interval frame selection resulted in satisfactory VR quality. The noted limitation was that when collecting images from different angles at a constant distance, images occasionally suffered from slight side drift. With the expected correction of such positioning drift, this Kinevo function would seem to possess significant anatomical educational and research value. Motorised movements of the microscope head have become significantly dampened so that there is little "wobble" upon braking to a new position. Still, such micro movements are present and dependent on the optimal balancing of the microscope. We were not able to reproduce multiline 3D QTVR that we had obtained previously with Zeiss MKM due to such small shifts upon breaking and used single line recordings with the Kinevo system instead [8].

Fluorescence

The introduction of a dedicated YELLOW 560 mode equipped on the operative microscope, has dramatically improved the intraoperative visualization of FNa (excitation and observation wavelength range of 460-500 nm and 540-690 nm respectively [19]). The YELLOW 560 fluorescence mode works by combining filtered wide-range visible light with an intense peak for FNa excitation, which is visible from the side as a "blue light", and special detection filter, which combines and balances the reflected visible spectrum light and yellow fluorescence light of FNa. FNa highlights cerebral vasculature [20] and various lesions including brain [21-23] and spinal cord [24] tumors. The results of a recent phase II clinical trial showed that a complete tumor resection can be achieved in 82.6% with FNa-guided technique [25].

The current study documents several important advances in fluorescence detection with the Kinevo, notably in FNa visualization. Previous versions of the YELLOW 560 filter did not display the red color well on the digital recordings. This was a particularly worrying disadvantage, as it did not allow for optimal intraoperative control of vessels in the shadowed region under the fluorescence modality. However, the intent for the FNa mode is that the surgeon can continue to operate without frequently switching to the white light mode. The Kinevo overcomes this limitation for digital imaging, as it provides a better discrimination between the fluorescent signal and the surrounding area by means of a significantly improved perception of brain and blood colors. Non-fluorescent tissues appeared much brighter and more natural in color with the Kinevo digital display and slightly brighter through oculars compared to the Pentero. The Kinevo allowed performance of nearly the entire tumor removal under the YELLOW 560 mode using the oculars for visualization, and the 3D visualization was noted to be similar in quality to the view through the oculars.

With regard to ICG visualization and INFRARED 800 mode, both systems resulted in relatively similar black-and-white ICG images. However, the Kinevo provided a novel augmented reality ICG overlay function [26], better image resolution, and improved post-processing workflow for quantification of the flow compared to the Pentero. Despite relatively infrequent use, FLOW 800 was shown to be clinically beneficial to detect ischemic brain regions [27], differentiation of the feeders and draining vessels in arteriovenous malformations [28] and quantitative evaluation of microanastomosis patency [29]. This study showed that ICG angiography was sensitive enough to assess blood flow even in small vessels less than 1 mm diameter with similar quantitative results.

The built-in video editing functions of the Kinevo allow improved processing of regular microscope video recording using white light or YELLOW 560 and BLUE 400 modes. Additionally, the Kinevo significantly supports improved video editing of INFRARED 800 recordings for quantification of the blood flow signal. Such processing was difficult using the Pentero.

Limitations

The clinical arm of this study is limited by its small size, the absence of a control group, multiple subjective measures, and concentration on cranial cases. The performed technology assessment was considered a pilot descriptive study of the potential utility of new, expanded, and improved functions and as a test for incorporation into the normal functionality of the neurosurgical operating room. Further studies on a larger cohort of patients are required to assess the impact of the technical advances or refinements on surgical outcomes. Different subspecialties within neurosurgery, e.g., cranial vs. spine surgery will likely find various aspects of the Kinevo system of different benefit, i.e., exoscopic function.  As well, use of the system by many surgeons will also form a basis with which to gage such a multi-function operative visualization platform. Interaction with navigation systems was not tested with the version of the Kinevo in the current study, but we believe the technological improvements in movement, tracking, and relatively adaptable operating system architecture will allow for exciting integration and display enhancements with image-guided surgical navigation platforms on the market or yet to be developed. Exact assessments of the usefulness of the system will need to be rigorously performed in the operating room with recreation in the laboratory for further study.

The new Kinevo YELLOW 560 filter, similarly to its previous versions, works only in combination with the BLUE excitation light. The new YELLOW 560 filter provides improved visualization of background, and such differences are especially noticeable in video recordings and when using the system as an exoscope, i.e., when viewing a digital image on the 3D screen. The "contamination" of pixels oversaturated with fluorescence is still present to some degree, but it is probably an uncorrectable drawback of any fluorescence-guidance surgery solution. In this regard, the new filters and camera represent a significant improvement for fluorescence visualization and viewing comfort for the surgeon compared to the previous version. Filter and excitation light cannot be turned on separately and always work in combination to avoid any damage to the surgeon's vision by unfiltered high-intensity reflection of excitation light.

Additionally, other surgical fields that use operative microscopes might potentially benefit from this robotic visualization platform. In today's medical economy in general, there is a need to expand the use of expensive devices for multidisciplinary use, since at least in the U.S. hospitals systems are the purchasers of such microscopes or surgical visualization platforms, including what may also be expensive long-term service contracts. Other surgical specialties may find such operative visualization systems useful, especially those that use exoscopic and fluorescence functions, and the robotic functions would seem to be attractive to most surgical specialties.

## Conclusions

Several advances have been introduced with a new robotic visualization platform, Kinevo. Compared to the Pentero, these improvements include improved intraoperative fluorescence visualization using YELLOW 560 mode, the integrated endoscopic micro-inspection tool, improved clarity of ocular imaging, and 3D image display on a 4K monitor enabling operating under exoscopic mode. Several novel robotic movement and control functions integrated into the microscope will further increase the maneuverability of the visualization platform. These new features could act to positively assist the surgeon and provide improved ergonomics and a greater level of intraoperative comfort, with a potential to increase the viewing quality neurosurgical procedures, including the illumination, visualization, and identification of pathology. New operational modes also allow significant impact for teaching and anatomy identification. Full incorporation with navigational system software will be the next step in the development and operational modes of such surgical microscope architecture. Although technologically exciting, these visualization platforms are becoming relatively complicated as they offer more functionality, which could lead to frustration or misuse. With the increasing number and complexity of functions of this new generation of surgical microscopes, it is apparent that surgeons should receive additional training in order to avoid underutilization and to avail themselves of the advantages of the numerous novel features.
